# On‐chip label‐free impedance‐based detection of antibiotic permeation

**DOI:** 10.1049/nbt2.12019

**Published:** 2021-02-02

**Authors:** Jaspreet Kaur, Hamed Ghorbanpoor, Yasin Öztürk, Özge Kaygusuz, Hüseyin Avcı, Cihan Darcan, Levent Trabzon, Fatma D. Güzel

**Affiliations:** ^1^ Department of Electrical and Electronic Engineering Yıldırım Beyazıt University Ankara Turkey; ^2^ Department of Biomedical Engineering Yıldırım Beyazıt University Ankara Turkey; ^3^ Department of Material Engineering Yıldırım Beyazıt University Ankara Turkey; ^4^ Biotechnology Application and Research Center Bilecik Şeyh Edebali University Bilecik Turkey; ^5^ Metallurgical and Materials Engineering Department Eskisehir Osmangazi University Eskisehir Turkey; ^6^ Cellular Therapy and Stem Cell Research Center (ESTEM) Eskisehir Osmangazi University Eskisehir Turkey; ^7^ AvciBio Research Group Eskisehir Osmangazi University Eskisehir Turkey; ^8^ Department of Molecular Biology and Genetics Bilecik Seyh Edebali University Bilecik Turkey; ^9^ Department of Mechanical Engineering Istanbul Technical University Istanbul Turkey; ^10^ Nanotechnology Research and Application Center – ITUnano Istanbul Technical University Istanbul Turkey

## Abstract

Biosensors are analytical tools used for the analysis of biomaterial samples and provide an understanding about the biocomposition, structure, and function of biomolecules and/or biomechanisms by converting the biological response into an electrical and/or optical signal. In particular, with the rise in antibiotic resistance amongst pathogenic bacteria, the study of antibiotic activity and transport across cell membranes in the field of biosensors has been gaining widespread importance. Herein, for the rapid and label‐free detection of antibiotic permeation across a membrane, a microelectrode integrated microfluidic device is presented. The integrated chip consists of polydimethylsiloxane based microfluidic channels bonded onto microelectrodes on‐glass and enables us to recognize the antibiotic permeation across a membrane into the model membranes based on electrical impedance measurement, while also allowing optical monitoring. Impedance testing is label free and therefore allows the detection of both fluorescent and non‐fluorescent antibiotics. As a model membrane, Giant Unilamellar Vesicles (GUVs) are used and impedance measurements were performed by a precision inductance, capacitance, and resistance metre. The measured signal recorded from the device was used to determine the change in concentration inside and outside of the GUVs. We have found that permeation of antibiotic molecules can be easily monitored over time using the proposed integrated device. The results also show a clear difference between bilayer permeation that occurs through the lipidic bilayer and porin‐mediated permeation through the porin channels inserted in the lipid bilayer.

## 1 INTRODUCTION

Antibiotic resistance of bacteria is a growing global problem. Especially, gram‐negative bacteria infections are the most challenging bacterial infections of our time [1, 2, 3]. Misuse of antibiotics and bacteria's ability to adapt themselves against almost all sorts of drugs have led to the issue of antibiotic resistance across the globe, and unavoidably the creation of ‘multi‐drug resistance’ in gram‐negative bacteria [4,5]. Among many factors, outer membrane channels/porins (Omp) that exist in the outer cell membrane of the bacteria are considered to play a crucial role in the resistance mechanism [6, 7, 8, 9, 10]. Knowing that Omps are nanoscale channels that allow the bilayer permeation by passive diffusion along the concentration gradient of hydrophilic molecules, nutrients, and drugs into the periplasmic space of the cell, one can imagine the importance of the channel as the first line of defence against any sorts of antibiotics. Channel modifications (due to environmental factors and mutations that cause a decrease or increase in porin diameter and number, change in polarity, and selectivity) or loss of cell envelope channels are the ways in which bacteria gain resistance to a specific antibiotic [11]. Thus, research into antibiotic permeation through these biological nanochannels is a key point in understanding the resistance mechanism that bacteria grow over and after the treatment period, as well as in developing novel rational antibiotics that can safely penetrate the cell wall and demonstrate their action against bacteria in the cell.

Monitoring the antibiotic permeation across a membrane in order to facilitate the treatment of resistant bacterial infections has therefore been investigated widely using different types of on‐chip biosensors [12, 13, 14, 15, 16, 17, 18, 19]. On‐chip biosensors are commonly made of advanced miniaturized diagnostics tools that are being used to detect and monitor the illnesses [20, 21, 22]. This advanced technology benefits from the advantages of lab on‐chip technologies consisting of sophisticated, but often very basic, microfluidic channel networks. Microfluidic systems have gone through many developments to perform complex and time‐consuming biological functions in a much effective and shorter time [23, 24, 25, 26]. These systems are also cost effective and automated while consuming small fluid volume [27, 28, 29].

**FIGURE 1:**
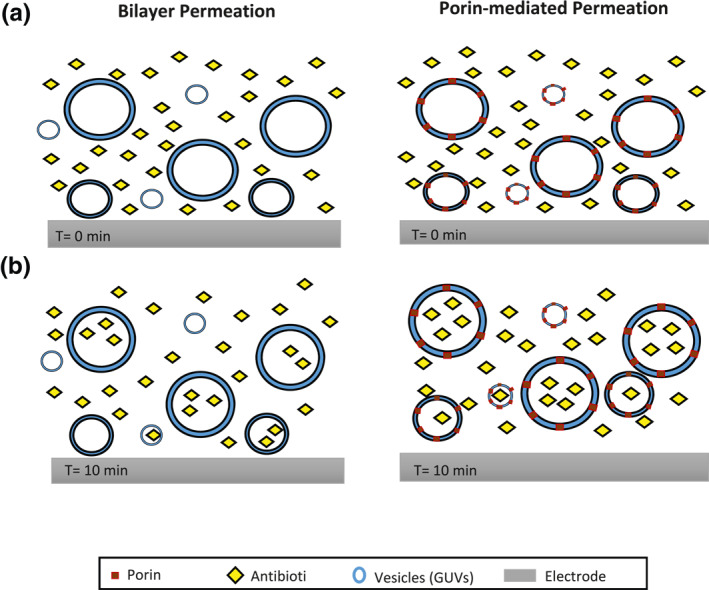
Schematic of permeation of the antibiotic molecules into the vesicles

Since antimicrobial resistance has become one of the global health problems of our time, the aim of our work was to use an integrated microfluidic device to check the rates of antibiotic permeation. When compared to the above studies, based on impedance testing using a precision inductance, capacitance, and resistance (LCR) metre, our device can perform on‐chip label‐free detection. The performance of the chip is easy, and the production is well known through classical semiconductor processing techniques. The novelty of our research arises from the use of impedance testing on the bilayer and porin‐mediated antibiotic permeation monitoring on‐chip for the very first time. The concept of the experiment is illustrated in Figure 1, where the concentration of antibiotic inside and outside of the vesicles, representing bacteria, varies over time and the change is observed using the electrode set‐up. Here mixing and the antibiotic permeation across a membrane occurs on‐chip. The integrated chip consists of a single microfluidic channel with two inlets for giant unilamellar vesicles (GUVs) and antibiotic, while the microelectrodes are made up of a thin layer of Ti and designed to be independently operating at certain durations. At the cross sections of the microfluidic channel and microelectrodes, the positive and negative electrodes are met with a particular distance. The length of the channels before each cross section is designed for the travel of the GUVs within a sufficient amount of time before each impedance measurement and allows for the measurement of the antibiotic permeation across a membrane over time, if exists. In conclusion, an alternative on‐chip label‐free susceptibility assay is presented, which would potentially be used for both autofluorescent and non‐autofluorescent antibiotics.

## 2 EXPERIMENTAL SECTION

### 2.1 Formation of giant unilamellar vesicles

GUVs were created by electroformation using an in‐house electroformation set‐up. We used 1,2‐diphytanoyl‐sn‐glycero‐3‐phosphocholine (DPhPC) lipid (Avanti Polar Lipids) to prepare GUVs. The physical properties of DPhPC create very high stability of planar bilayers. DPhPC has pKa of 2.1 and 13.9; therefore, it remains neutral in a wide range of pH. Moreover, it does not have a phase transition from –120 to 80°C [30]. 60 μl of 5 mg⋅ml^−1^ of DPhPC lipid in chloroform was spread on the conducting surface of an indium titanium oxide (ITO)‐coated glass slide within an o‐ring. The chloroform was evaporated for 10 min in a desiccator, followed by the addition of 600 μl of the 200 mM sucrose within the o‐ring. A sandwich structure was made with another ITO‐coated slide (conducting surfaces facing each other). This was placed in the set‐up whereupon electroformation proceeds in three steps: (i) the a/c voltage increases linearly from 0 to 3 Vpp at 5 Hz in 5 min. (ii) The voltage stays at 3 Vpp and 5 Hz for 2 h. (iii) The voltage decreases linearly to 0 V at 5 Hz in 5 min. The electroformation was carried out at 37.5°C. The vesicles were stored at 20°C and used within a week. For the preparation of porin‐loaded GUVs, 5.5 ng purified stock of OmpF was diluted in 1% solution of octyl‐polyoxyethylene (OPOE) prepared in 1 ml of deionized water. 2.5 μl diluted OmpF solution was then added to 500 μl GUVs and incubated over 1 h. Bio beads were later added to the incubated solution to remove the detergent, followed by a second incubation in the refrigerator. Afterwards, the pro‐GUVs were separated from the bio beads to use in the following experiments. The size distribution of the GUVs were carried out using ImageJ.

### 2.2 Cloning, expression, and purification of *E. coli* OmpF porin protein

Primers of the *ompF* gene were designed in accordance with the pLATE51 vector in the aLICator LIC Cloning and Expression Kit 2 (ThermoFisher Scientifics K1251). The polymerase chain reaction (PCR) product of the *Escherichia coli ompF* porin gene which was amplified by colony PCR using specific primers was cloned into the pLATE51 vector. The plasmid was then transformed into wild‐type *E. coli* W3110 strain. Transformant *E. coli* transferred to 100 ml LB broth, incubated at 160 rpm at 37°C until the OD_600_ value reached 0.6–0.8 absorbance. After that, a final concentration of 0.5 mM Isopropyl β‐ d‐1‐thiogalactopyranoside was added to the culture and 6‐h shaking incubation was continued to induce the plasmid. After incubation, 100 ml bacterial cells were centrifuged at 12,000 rpm for 5 min at 4°C. After that, protein purification was carried out according to the procedure of the Macherey‐Nagel Protino^®^ Ni‐NTA Agarose kit. Purified proteins were analysed by western blot using anti‐Histag antibody [31, 32, 33].

### 2.3 Preparation of aqueous solutions

**FIGURE 2:**
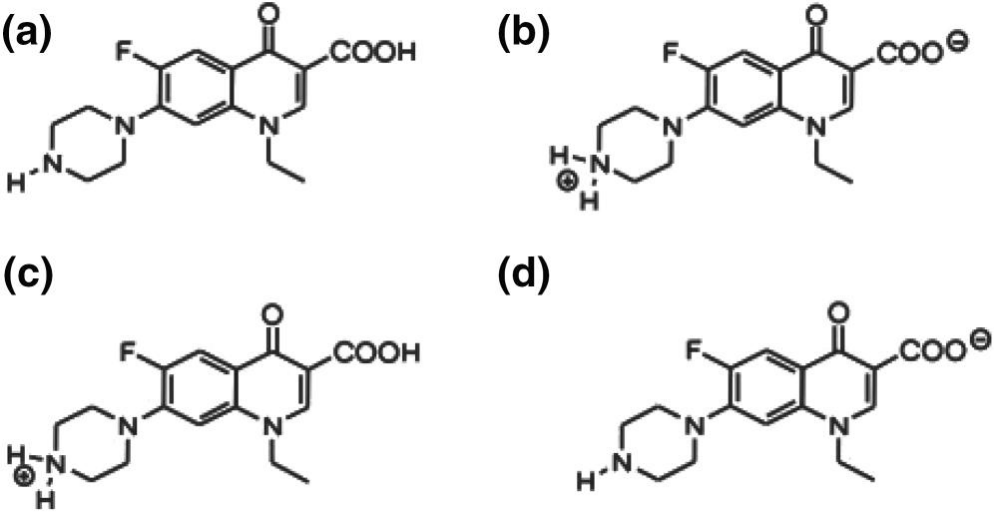
Different ionized and non‐ionized forms of norfloxacin

200 mM of sucrose solution was prepared in deionized water (DI) water (pH 7). To adjust the pH of 2 mM of norfloxacin prepared in DI water, tris(hydroxymethyl)aminomethane and hydrochloric acid were used. For each experiment, daily prepared fresh GUVs were used to maintain consistency. Figure 2 displays different ionized and non‐ionized forms of norfloxacin. Depending on the pH, four forms in a solution can be obtained: (a) neutral, (b) zwitterion, (c) cation, and (d) anion. Neutral and zwitterion structures are formed at pH 7, while cation and anion forms are obtained at pH 5 and pH 9, respectively [34].

### 2.4 Chip design and fabrication

The fabrication process is divided into three stages: (a) polydimethylsiloxane (PDMS)‐made microfluidic chips were fabricated using classical lithography [26]. The fabricated device has a network of microfluidic channels that are 200 μm wide and 25 μm high. The total length of the network from the T junction to the outlet reservoir is approximately 380 mm, where the width of the horizontal channels is 200 µm and the vertical channel width is 300 µm. The channel design has two inlets for receiving a solution containing GUVs and antibiotic:—in this case, norfloxacin—and an outlet for collection of norfloxacin‐loaded GUVs.

(b) Microelectrode arrays were first designed in AUTOCAD and then transferred to CORELDRAW for high resolution. The pattern masks were saved as .pdf format and printed on acetate paper at Çözüm Tanıtım, Turkey. To allow easy and precise integration, alignment markers were also designed on both the microfluidic chip and the microelectrode chip with flexibility of 50 μm. The electrode width is 100 µm, the gap between electrode fingers which comprises positive and negative electrodes is 50 µm, and the total length of each electrode is 10.975 mm.

Microscope slides were cut in half and the prepared substrates were cleaned first in 1 M KOH solution for 10 min, and then in acetone for another 10 min using an ultrasonic cleaner. The substrates were then rinsed with isopropyl alcohol, DI‐water, and blow‐dried with nitrogen gas. After cleaning, the substrates were coated with 2.5 μl positive photoresist AZ‐9620 using a single substrate spin processor. Before exposure, the photoresist coating was soft baked for 2 min 45 s at 110°C. The photoresist‐coated substrates were placed under the pattern mask with scotch tape and exposed to an in‐house developed UV light source for 70 s. After exposure, the photoresist on the substrates was developed in tetraethylammonium hydroxide (TEAH) for 2 min 30 s and rinsed with DI water. The glass templates were loaded into a sputtering chamber to deposit a 200‐nm thin film of titanium at a rate of 7 nm/min. After deposition, the substrates were kept in vacuum for an hour before proceeding to the lift‐off process.

**FIGURE 3:**
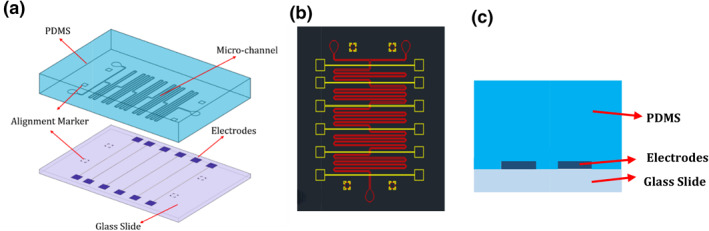
a) 3D view of the integrated chip. (b) 2D view represents the cross section of electrodes and microchannels. (c) Schematic of the cross section of the integrated chip

(c) Microfluidic chip was bonded to the Ti‐on glass microelectrodes after plasma exposure of the two surfaces. The device was then placed on a hot plate at 110°C for 120 min to enhance the adhesion. Figure 3a shows the 3D view of the integrated device before the integration. The schematic of the integrated device is shown in Figure 3b,c.

### 2.5 Impedance measurements

The impedance measurements were measured with a precision LCR metre (HM8118 LCR Bridge/Metre, Germany) [5,[35]. The electrical connections to the LCR metre were made by alligator clips to the copper wires. Copper wires were attached to the electrical pads on the glass chip using conductive silver epoxy and the chip was then kept on a hotplate at 125°C for an hour to harden the epoxy. All of the wires and the chip itself were fixed onto the focal plane of the microscope with a tape. A 1‐mm plastic tubing was inserted into the chip inlets. A micropump was used to inject the GUVs and antibiotic solution to the respective inlets of the microchip. All experiments were repeated four times at 1.5 V and 100 kHz. The measurements were independently taken at the positive and negative electrodes aligned across each other – six measurement points.

### 2.6 Statistical analysis

Statistical analysis on all results was performed using paired sample *t*‐test in SPSS software. Statistical significance threshold was set at 0.05 (*p* < 0.05). Error bars represent a standard deviation of the mean (*n* = 4).

## 3 RESULTS AND DISCUSSION

Herein, the authors aimed to investigate the bilayer permeation using GUVs without porins and porin‐mediated permeation using pro‐GUVs loaded with OmpF porins of *E. coli*. Bilayer permeation occurs through the lipid bilayer, whereas porin‐mediated permeation refers to the transport of antibiotics through OmpF porin in the lipid bilayer and is expected to lead to higher permeation rates due to the natural diffusion pathway created via the porin channels. In order to imitate the diffusion pathway in the bacteria, GUVs were used as a model membrane, because working with bacteria with only a specific porin existing in the cell outer membrane would require extensive microbiology work. Here GUVs were fabricated using electroformation and the technique is easy to implement for the formation of different GUVs with different lipids in a different size. DPhPC lipids were used herein and the size distribution analysis of the GUVs showed that approximately 95% of the GUVs were under 30 µm in size.

**FIGURE 4:**
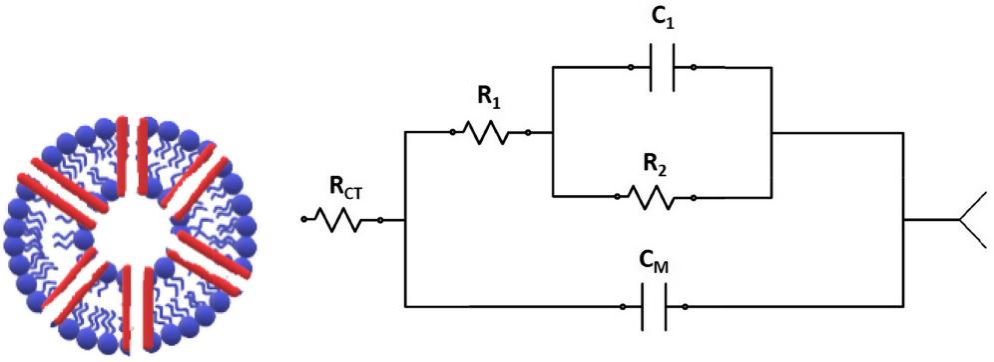
Left: Schematic of the lipid bilayer. Right: Simple electric model of the membrane

As for the permeability testing, an LCR metre was chosen to measure the impedance. Impedance testing has been previously reported to be sensitive to the cell type that could be stimulated by different chemicals or drugs [36,[37]. Likewise, we hypothesized that the uptake of antibiotics into the GUVs could be monitored by a simple impedance testing because the outer membrane of GUVs in solution comprises an RC circuit [38, 39, 40] (Figure 4).

The ionic media outside and inside of the GUVs constitute a conducting circuit, while the lipid bilayer of the GUVs acts as a capacitor separating two ionic media. Here the charge separation which leads to a potential difference across the lipid bilayer can be related to the concentration through a simplified Nernst equation:

$$V=\frac{RT}{zF}\ln\frac{C_{\text{o}}}{C_{\text{i}}}$$

where *V* is the voltage, *R* is the gas constant, *T* is the temperature, *z* is the valence, *F* is the Faraday constant, and *C* is concentration. It means that *V* across the membrane is in equilibrium with the concentration gradient established by the outside ionic concentration (*C*_o_) and inside ionic concentration (*C*_i_) inside where the net ionic flux is zero. In other words, the concentration can then be related to the impedance through the voltage difference [41]:

$$Z=\frac{V}{I}=\frac{RT}{zF}\ln\frac{C_{\text{o}}}{C_{\text{i}}}/I$$

It is evident from the equation that if the ionic concentration inside the vesicle increases, then the impedance at particular pH decreases. In this context, the measured impedance value in our experiment would imply that the concentration changes in and outside would mean a change in the impedance and then could be used as a determining factor for the permeation rates.

In line with this discussion, we measured the impedance values of the solution containing GUVs and the antibiotic at different pH. GUVs and antibiotics were introduced via two independent inlets on the chip, as previously shown in Figure 3. While the solution moved through the channel at a flow rate of 0.5 μl/min, the impedance values were measured at each electrode over a certain period. It is assumed that measurement after the arrival of the solution to the measurement region—electrode junctions—within 2‐, 4‐, 6‐, 8‐, and 10‐min intervals would be sufficient to monitor the permeabilities. We placed 12 (6 positive and negative each) electrodes underneath the channel which comprise 6 junctions. That is to say that there was a total of six junctions (shown in Figure 3) where impedance was measured to ensure the change in the permeability of the antibiotic over time. The microfluidic channel is 360 mm long and allows the travel of the solution within about 7 min. Within minutes, the antibiotics penetrate the GUVs through the diffusion channel [42, 43, 44]. Measurements were taken at different pH to ensure the capture of permeation rates at different conditions and to be able to compare. Norfloxacin was chosen as a reference antibiotic due to its fluorescence properties, which would then enable us to double monitor the permeability both electrochemically and optically. Permeation rates were calculated from the impedance changes over time using the equation ‘*Z*_final_ − *Z*_initial_/*Z*_final_ × 100’.

**FIGURE 5:**
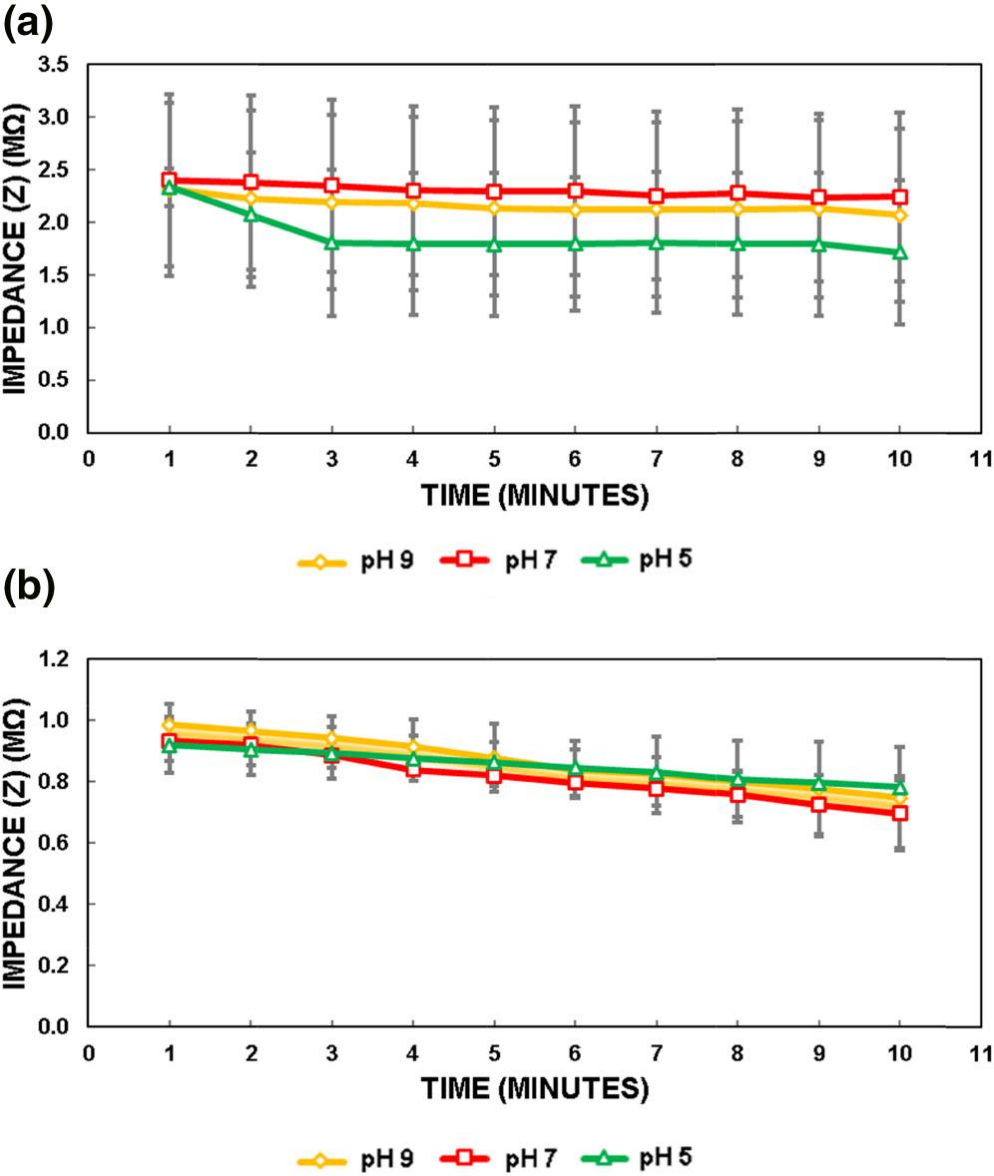
Impedance results for bilayer and porin‐mediated permeation at different pH: (a) bilayer permeation; (b) porin‐mediated permeation. Error bars represent standard error of the mean (*n* = 4), *p* > 0.05

Figure 5 shows the impedance results for bilayer and porin‐mediated permeation using GUVs and pro‐GUVs at different pH. As clearly seen from Figure 5a,b, there is a drop in the impedance values in both cases, and the decrease is much sharper when using pro‐GUVs, in the order of 0.3 MΩ. In particular, in bilayer permeation testing, the permeation rates were 17%, 7%, and 11%, at pH 5, 7, and 9, respectively. In porin‐mediated permeation testing, where new chips were used, the error bars were significantly lowered. The permeation rates for the porin‐mediated permeation were found to be 36%, 34%, and 31% at pH 5, 7, and 9, respectively. The presence of the porin resulted in significant uptake of the antibiotics obviously, and this caused a considerable increase in the permeation rates. It is interesting to find that the effect of pH was more observable in the absence of the porins in the lipid bilayer of the GUVs. However, there is no significant difference between data (the *t*‐test demonstrates that the *p*‐value is higher than 0.05 [>0.05]). In contrast, the pH did not play a significant role in the porin‐mediated permeation. Norfloxacin at pH 7 demonstrates zwitterion, whereas, at pH 5 and pH 9, the antibiotic molecules become positively and negatively charged [34]. The control experiments lacking norfloxacin were also performed and found that the change in impedance is in the order of 10 kOhm and is therefore negligible compared to the MOhm change obtained after the addition of norfloxacin. Though the pH effect remains questioned and requires further studies, probably with different antibiotics, the integrated device was capable of detecting the changes over time and proves the proof of concept to be a candidate for the label‐free on‐chip antibiotic permeation testing, an alternative to on‐chip fluorescence‐based assays [43].

## 4 CONCLUSION

Herein, a detailed vesicle permeation of antibiotics is investigated on an integrated chip. Electroformation‐generated vesicles mimic a living cell's cell membrane, whereas impedance measurements are a way of assessing label‐free permeation rates over time. A PDMS‐made microfluidic chip integrated onto a titanium electrode array was designed and produced to develop the label‐free on‐chip assay. As a proof of concept, the hybrid device was then used for the bilayer and porin‐mediated permeation testing. Experimental findings show that the device is effective in understanding the permeation rates using impedance changes over predetermined times with respect to concentrations in and out of the vesicles. Future studies should be directed towards the single or multiplexed testing of other fluoroquinolones and amino acids with different chemical properties such as charge, in order to further investigate the capacity of the integrated chip. We conclude that in various evaluations of in vitro antibiotic permeation platforms, whether the drug is autofluorescent or non‐autofluorescent; this flexible and robust concept and the hybrid device can be widely used.

## References

[1] Levy, S.B. : Antibiotic resistance – the problem intensifies. Adv. Drug Deliv Rev. 57, 1446–1450 (2005) 15949867 10.1016/j.addr.2005.04.001

[2] Alekshun, M.N. , Levy, S.B. : Molecular mechanisms of antibacterial multidrug resistance. Cell. 128, 1037–1050 (2007) 17382878 10.1016/j.cell.2007.03.004

[3] Arias, C.A. , Murray, B.E. : Antibiotic‐resistant bugs in the 21st century – a clinical super‐challenge. N. Engl. J. Med. 360(5), 439–443 (2009) 19179312 10.1056/NEJMp0804651

[4] Norouz Dizaji, A. , et al.: In vivo imaging/detection of MRSA bacterial infections in mice using fluorescence labelled polymeric nanoparticles carrying vancomycin as the targeting agent. J. Biomater. Sci. Polym. Ed. 31(3), 293–309 (2020) 31762403 10.1080/09205063.2019.1692631

[5] Kaur J. , et al.: Integrated microfluidic chip development for the quantification of antibiotic permeability rates through bacteria cell wall. https://www.researchgate.net/publication/334172928_Integrated_Microfluidic_Chip_Development_for_the_Quantification_of_Antibiotic_Permeability_Rates_through_Bacteria_Cell_Wall. Accessed October 2020

[6] Darcan, C. , Ozkanca, R. , Flint, K.P. : Survival of nonspecific porin‐deficient mutants of Escherichia coli in Black Sea water. Lett. Appl. Microbiol. 37(5), 380–385 (2003) 14633108 10.1046/j.1472-765x.2003.01418.x

[7] Dupont, M. , et al.: An early response to environmental stress involves regulation of OmpX and OmpF, two enterobacterial outer membrane pore‐forming proteins. Antimicrob. Agents Chemother. 51(9), 3190–3198 (2007) 17606680 10.1128/AAC.01481-06 PMC2043185

[8] Yao, Z. , et al.: Quantitative proteomics reveals antibiotics resistance function of outer membrane proteins in aeromonas hydrophila. Front Cell. Infect. Microbiol. 8, 390 (2018) 30460208 10.3389/fcimb.2018.00390 PMC6232253

[9] Viveiros, M. , et al.: Antibiotic stress, genetic response and altered permeability of E. coli. PLoS One. 2(4), e365 (2007) 17426813 10.1371/journal.pone.0000365 PMC1838523

[10] Masi, M. , Pagès, J.‐M. : Structure, function and regulation of outer membrane proteins involved in drug transport in Enterobactericeae: the OmpF/C – TolC Case. Open Microbiol. J. 7(1), 22–33 (2013) 23569467 10.2174/1874285801307010022 PMC3617542

[11] Pagès, J.M. , James, C.E. , Winterhalter, M. : The porin and the permeating antibiotic: A selective diffusion barrier in Gram‐negative bacteria. Nat. Rev. Microbiol. 6, 893–903 (2008) 18997824 10.1038/nrmicro1994

[12] Guzel, F.D. , Citak, F. : Development of an on‐chip antibiotic permeability assay with single molecule detection capability. IEEE Trans. Nanobiosci. 17(2), 155–160 (2018) 10.1109/TNB.2018.2809592 29870339

[13] Cama, J. , et al.: Quantification of fluoroquinolone uptake through the outer membrane channel OmpF of Escherichia coli. J. Am. Chem. Soc. 137(43), 13836–13843 (2015) 26478537 10.1021/jacs.5b08960

[14] Sun, H. , et al.: Reliable and reusable whole polypropylene plastic microfluidic devices for a rapid low‐cost antimicrobial susceptibility test. Lab Chip. 19(17), 2915–2924 (2019) 31369010 10.1039/c9lc00502a

[15] Dai, J. , Hamon, M. , Jambovane, S. : Microfluidics for antibiotic susceptibility and toxicity testing. Bioengineering. 3 (2016) 10.3390/bioengineering3040025 PMC5597268 28952587

[16] Lee, C.R. , et al.: Strategies to minimize antibiotic resistance. Int. J. Environ. Res. Public Health. 10, 4274–4305 (2013) 24036486 10.3390/ijerph10094274 PMC3799537

[17] Łapińska, U. , et al.: Bacterial ageing in the absence of external stressors. Philos. Trans. R. Soc. B Biol. Sci. 374(1786), 20180442 (2019) 10.1098/rstb.2018.0442 PMC6792439 31587633

[18] Cama, J. , et al.: Single‐cell microfluidics facilitates the rapid quantification of antibiotic accumulation in Gram‐negative bacteria. Lab Chip. 20(15), 2765–2775 (2020) 32613221 10.1039/d0lc00242a PMC7953842

[19] Bamford, R.A. , et al.: Investigating the physiology of viable but non‐culturable bacteria by microfluidics and time‐lapse microscopy. BMC Biol. 15(1), 121 (2017) 29262826 10.1186/s12915-017-0465-4 PMC5738893

[20] Dumay, A.C.M. , Blank, J.L.T. : Healthcare prosumerism. In: Bos, L. , Carroll, D. , Kun, L. , Marsh, A. , Roa, L. (eds.) Future Visions on Biomedicine and Bioinformatics, vol. 1, pp. 43–52. Springer, Heidelberg (2010)

[21] Lunenfeld, B. , Stratton, P. : The clinical consequences of an ageing world and preventive strategies. Best Pract. Res. Clin. Obstet. Gynaecol. 27(5), 643–659 (2013) 23541823 10.1016/j.bpobgyn.2013.02.005 PMC3776003

[22] Dogan Guzel, F. , Avci, H. : Fabrication of nanopores in an ultra‐thin polyimide membrane for biomolecule sensing. IEEE Sens. J. 18(7), 2641–2646 (2018)

[23] Neužil, P. , et al.: Revisiting lab‐on‐a‐chip technology for drug discovery. Nat. Rev. Drug Discov. 11, 620–632 (2012) 22850786 10.1038/nrd3799 PMC6493334

[24] Rothbauer, M. , Zirath, H. , Ertl, P. : Recent advances in microfluidic technologies for cell‐to‐cell interaction studies. Lab Chip. 18, 249–270 (2018) 29143053 10.1039/c7lc00815e

[25] Cui, P. , Wang, S. : Application of microfluidic chip technology in pharmaceutical analysis: a review. J. Pharm. Anal. 9(4), 238–247 (2019) 31452961 10.1016/j.jpha.2018.12.001 PMC6704040

[26] Güzel, F.D. , Miles, B. : Development of in‐flow label‐free single molecule sensors using planar solid‐state nanopore integrated microfluidic devices. Micro. Nano Lett. 13(9), 1352–1357 (2018)

[27] Gupta, S. , et al.: ‘Lab‐on‐chip technology: a review on design trends and future scope in biomedical applications. Int. J. Bio‐Science Bio‐Technol. 8(5), 311–322 (2016)

[28] Wu, J. , et al.: Lab‐on‐chip technology for chronic disease diagnosis NPJ. Digit. Med. 1(1) (2018) 10.1038/s41746-017-0014-0 PMC6550168 31304292

[29] Mark D. , et al.: Microfluidic lab‐on‐a‐chip platforms: requirements, characteristics and applications. Chemical Society Reviews. 39(3), 1153(2010) 20179830 10.1039/b820557b

[30] Batishchev, O.V. , Indenbom, A.V. : Alkylated glass partition allows formation of solvent‐free lipid bilayer by Montal‐Mueller technique. Bioelectrochemistry. 74(1), 22–25 (2008) 18378502 10.1016/j.bioelechem.2008.02.002

[31] Wang, X. , et al.: Escherichia coli outer membrane protein F (OmpF): an immunogenic protein induces cross‐reactive antibodies against Escherichia coli and Shigella. AMB Express. 7(1) (2017) 10.1186/s13568-017-0452-8 PMC5517391 28728309

[32] Kaufmann, S.H. , Ewing, C.M. , Shaper, J.H. : The erasable Western blot. Anal. Biochem. 161(1), 89–95 (1987) 3578791 10.1016/0003-2697(87)90656-7

[33] Mahmood, T. , Yang, P.C. : ‘Western blot: technique, theory, and trouble shooting. N. Am. J. Med. Sci. 4(9), 429–434 (2012) 23050259 10.4103/1947-2714.100998 PMC3456489

[34] Barret, R. : Importance and evaluation of lipophilicity. In: Barret, R. Therapeutical Chemistry, pp. 53–78. Elsevier (2018)

[35] Kaur J. , et al.: Label‐free on‐chip antibiotic permeability assay. https://www.researchgate.net/publication/336533364_o-42_Label-free_On-chip_Antibiotie_Permeability_Assay. Accessed October 2020

[36] Altinagac, E. , Taskin, S. , Kizil, H. : Single cell array impedance analysis for cell detection and classification in a microfluidic device. In: Proceedings of the 10th International Joint Conference on Biomedical Engineering Systems and Technologies (BIOSTEC 2017), 49‐53

[37] Guzel, F.D. , et al.: Label‐free molecular detection of antibiotic susceptibility for Mycobacterium smegmatis using a low cost electrode format. Biotechnol. Appl. Biochem. (2020) 10.1002/bab.2037 32975308

[38] Miles, F.A. : The nerve impulse. In: Miles, F.A. Excitable Cells, pp. 38–70.Elsevier (1969)

[39] Diamanti, E. , et al.: Gramicidin ion channels in a lipid bilayer supported on polyelectrolyte multilayer films. An electrochemical impedance study. Soft Matter. 13(47), 8922–8929 (2017) 29143830 10.1039/c7sm01539a

[40] Amin, M. , Dey, P.P. , Badkoobehi, H. : A complete electrical equivalent circuit model for biological cell. https://www.researchgate.net/publication/262177526_A_complete_electrical_equivalent_circuit_model_for_biological_cell. Accessed June 2020

[41] Miles, F.A. : The nerve impulse. In: Miles, F.A. Excitable Cells, pp. 38–70. Elsevier (1969)

[42] Cama, J. , Javer, A. , Keyser, U.F. : A label‐free microfluidic assay to quantitatively study antibiotic diffusion through lipid membranes. Lab Chip. 14, 2303–2308 (2014) 24825393 10.1039/c4lc00217b

[43] Cama J. , et al. Quantification of Fluoroquinolone uptake through the outer membrane channel OmpF of Escherichia coli. J. Am. Chem. Soc. 137(43), 13836–13843 (2015) 26478537 10.1021/jacs.5b08960

[44] Cama J. , et al.: Direct optofluidic measurement of the lipid permeability of fluoroquinolones. Scientific Reports 6(1), (2016) 10.1038/srep32824 PMC5015079 27604156

